# µ-Conotoxins Targeting the Human Voltage-Gated Sodium Channel Subtype Na_V_1.7

**DOI:** 10.3390/toxins14090600

**Published:** 2022-08-30

**Authors:** Kirsten L. McMahon, Hue N. T. Tran, Jennifer R. Deuis, David J. Craik, Irina Vetter, Christina I. Schroeder

**Affiliations:** 1 Institute for Molecular Bioscience, The University of Queensland, Brisbane, QLD 4072, Australia; 2 The School of Pharmacy, The University of Queensland, Woolloongabba, QLD 4102, Australia; 3 Center for Cancer Research, National Cancer Institute, National Institutes of Health, Frederick, MD 21702, USA

**Keywords:** µ-conotoxins, voltage-gated sodium channels, structure-activity relationships, disulfide-rich peptides, Cys frameworks

## Abstract

µ-Conotoxins are small, potent, peptide voltage-gated sodium (Na_V_) channel inhibitors characterised by a conserved cysteine framework. Despite promising in vivo studies indicating analgesic potential of these compounds, selectivity towards the therapeutically relevant subtype Na_V_1.7 has so far been limited. We recently identified a novel µ-conotoxin, SxIIIC, which potently inhibits human Na_V_1.7 (hNa_V_1.7). SxIIIC has high sequence homology with other µ-conotoxins, including SmIIIA and KIIIA, yet shows different Na_V_ channel selectivity for mammalian subtypes. Here, we evaluated and compared the inhibitory potency of µ-conotoxins SxIIIC, SmIIIA and KIIIA at hNa_V_ channels by whole-cell patch-clamp electrophysiology and discovered that these three closely related µ-conotoxins display unique selectivity profiles with significant variations in inhibitory potency at hNa_V_1.7. Analysis of other µ-conotoxins at hNa_V_1.7 shows that only a limited number are capable of inhibition at this subtype and that differences between the number of residues in loop 3 appear to influence the ability of µ-conotoxins to inhibit hNa_V_1.7. Through mutagenesis studies, we confirmed that charged residues in this region also affect the selectivity for hNa_V_1.4. Comparison of µ-conotoxin NMR solution structures identified differences that may contribute to the variance in hNa_V_1.7 inhibition and validated the role of the loop 1 extension in SxIIIC for improving potency at hNa_V_1.7, when compared to KIIIA. This work could assist in designing µ-conotoxin derivatives specific for hNa_V_1.7.

## 1. Introduction

Marine cone snail venom comprises a diverse mixture of disulfide-rich ion channel modulators called conotoxins [1]. µ-Conotoxins, characterised by a conserved cysteine framework (i.e., CC–C–C–CC), potently inhibit voltage-gated sodium (Na_V_) channels through a pore blockage mechanism [2]. Although humans express nine different Na_V_ channel subtypes, µ-conotoxins have a preference for TTX-sensitive (TTX-s) subtypes, Na_V_1.1–1.4, 1.6 and Na_V_1.7, over TTX-resistant (TTX-r) subtypes Na_V_1.5, 1.8 and 1.9 [3,4,5]. Of particular interest is their inhibitory potency at Na_V_1.7, as this subtype has therapeutic potential for the treatment of pain [6,7]. However, the translation from hit to drug lead is hindered by the lack of specificity of µ-conotoxins for Na_V_ channel subtypes. Given the essential role of Na_V_ channels in the generation and propagation of action potentials, off-target inhibition of Na_V_ channels could lead to serious unwanted side-effects. Therefore, identifying µ-conotoxin residues contributing to potent and selective inhibition of human (h)Na_V_1.7 channels would aid in the development of µ-conotoxin-derived therapeutics for diseases such as chronic pain.

Of the 22 µ-conotoxins characterised to date, only a few display potent inhibition of hNa_V_1.7 [4,8]. We recently reported the discovery of SxIIIC, a novel µ-conotoxin that inhibits hNa_V_1.7 with nanomolar potency, though non-selectively [9]. SxIIIC has high sequence homology with two other µ-conotoxins, SmIIIA and CnIIIC [10,11], both of which have been evaluated against mammalian Na_V_ channels, but only CnIIIC was characterised at human isoforms (hNa_V_1.7 IC_50_ 485 ± 94 nM) [4]. KIIIA is the only other µ-conotoxin reported to potently inhibit hNa_V_1.7 (IC_50_ 97–363 nM), although also in a non-selective fashion [8]. Several studies have used KIIIA to investigate residues essential for Na_V_ inhibition and identified several within the C-terminal α-helix of the peptide [8,12,13]. Those studies resulted in approaches for µ-conotoxin optimisation, including isolating the helical residues and downsizing KIIIA [14,15,16,17,18,19]. For example, Khoo and colleagues stabilised the KIIIA pharmacophore with a lactam helix, and while the selectivity profile across Na_V_ channel subtypes remained unaffected, the stabilised helix only yielded micromolar inhibitory potency [14]. In another example, a peptidomimetic based on the KIIIA pharmacophore residues was designed by Brady et al., but only showed micromolar inhibition of Na_V_1.7 (species not reported) [15]. Other researchers have opted for a combination approach and designed ‘mini’ and ‘midi’ variants by combining two µ-conotoxins [16,17], or combining µ-conotoxins and spider toxins [18,19]; however, these too showed limited, non-selective inhibitory potency at hNa_V_1.7.

In the present study, we used electrophysiology assays to characterise the hNa_V_ channel selectivity profiles of µ-conotoxins closely related to SxIIIC, including SmIIIA and KIIIA for Na_V_1.7 (neuronal and pain related) over Na_V_1.4 (muscle) sodium channel. Through structure-activity relationship analysis of hNa_V_1.7-inhibiting µ-conotoxins, we discovered point mutations that affect potency and selectivity at hNav1.7. These findings could lead to the development of subtype-selective µ-conotoxin derivatives with potential for therapeutic development.

## 2. Results

### 2.1. Oxidation of Native µ-Conotoxins and Analogues

SxIIIC analogues including [Δ1,2]SxIIIC, [R16A]SxIIIC, [R16H]SxIIIC, [R16Q]SxIIIC, [D17A]SxIIIC, [R20A]SxIIIC, [R20E]SxIIIC, [R20H]SxIIIC, [R20Q]SxIIIC, [R20W]SxIIIC were produced using thermodynamic folding as the majority of these peptides produced one major isomer during folding as observed for native SxIIIC, and this major isomer was used for our electrophysiology studies (Figure S2). These peptides were not analysed further using NMR or high-resolution MS (HR-MS) for disulfide bond connectivity confirmation due to the similarities in RP-HPLC chromatogram compared to native SxIIIC suggesting native disulfide connectivity. SmIIIA, the KIIIA analogue [loop1R]KIIIA and SxIIIC analogues ([Δ7,8]SxIIIC, [Δ6–9]SxIIIC and [G8R]SxIIIC) were produced using regioselective folding as they did not form one major isomer during thermodynamic folding conditions (Figure S3). SxIIIC analogues [Δ1,2:R16H]SxIIIC, [Δ1,2:R20A]SxIIIC, [R16H:R20A]SxIIIC and [Δ1,2:R16H:R20A]SxIIIC) produced two main peaks during RP-HPLC analysis following thermodynamic folding (Figure S4) and both peaks were evaluated using 1D ^1^H NMR (Figure S5) and compared to the NMR spectrum of native SxIIIC. When comparing the NMR spectrum of the SxIIIC peak 1 analogues (blue arrow in Figure S4), several additional or missing peaks were noted in amide region compared to the NMR spectrum of native SxIIIC. The 1D ^1^H NMR of the second peak more closely resembled native SxIIIC suggesting native disulfide bond conformation (Figure S5), leading us to use the second peak (red arrow in Figure S4) for electrophysiology inhibitory potency evaluation.

### 2.2. Selectivity of µ-Conotoxins for hNa_V_ Channel Subtypes

**Figure 1:**
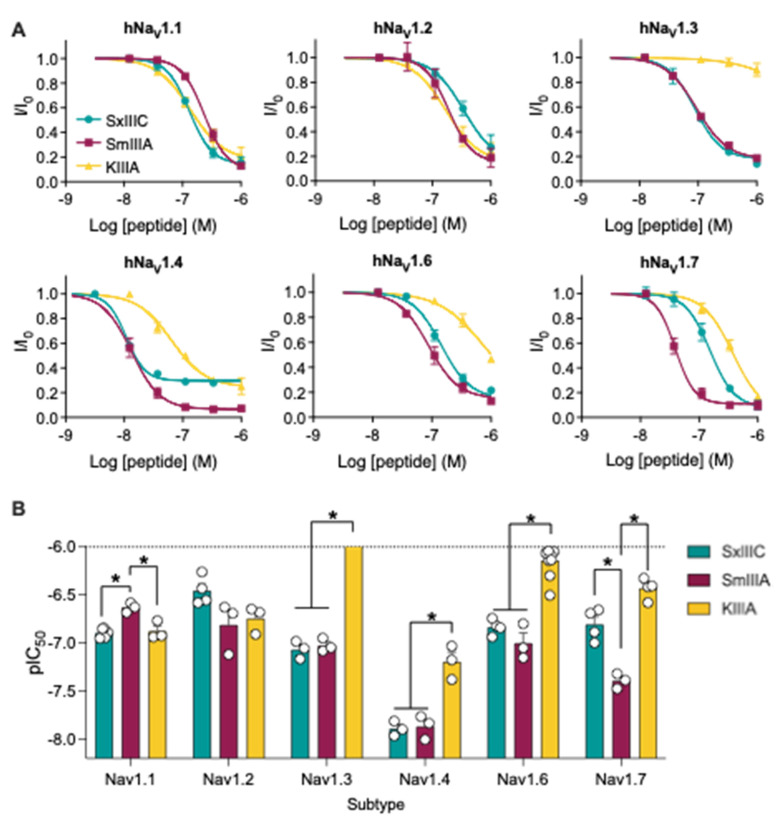
**Comparison of pharmacological inhibitory potency of µ-conotoxins SxIIIC, SmIIIA and KIIIA as assessed by whole-cell patch-clamp electrophysiology across hNa_V_1.1–1.7/β1 overexpressed in HEK cells**. (**A**) Concentration-response curves and (**B**) comparison of µ-conotoxin potency across Na_V_ channel subtypes. SxIIIC data from [9]. Data are presented as mean ± SEM with individual data points (*n* = 3–10). Statistical significance was calculated by ordinary one-way ANOVA analysis and Tukey’s multiple comparisons test (* *p*-value < 0.05).

µ-Conotoxin SmIIIA has been reported to only weakly inhibit mammalian Na_V_1.7 [20,21], despite sharing ~90% sequence homology with the recently described non-selective hNa_V_1.7 inhibitor SxIIIC [9]. KIIIA has also been shown to inhibit hNa_V_1.7, albeit at various potencies probably due to the use of different species of Na_V_1.7 and different recording systems [8,22]. To gain a comprehensive understanding of potency and selectivity of SmIIIA and KIIIA across human Na_V_ channel subtypes, we assessed the peptides by automated whole-cell patch-clamp electrophysiology in HEK293 cells expressing hNa_V_1.1–1.7/β1 and CHO cells expressing hNa_V_1.8/β3 and compared inhibitory potency with our results for SxIIIC in the same cell lines. Concentration-response curves (Figure 1A) were used to determine IC_50_ values for the µ-conotoxins at each channel subtype (Table 1). Comparison of IC_50_ values revealed differences in subtype selectivity between the three µ-conotoxins (Figure 1B). All three µ-conotoxins most potently inhibited hNa_V_1.4, with SxIIIC (IC_50_ 15 ± 11 nM) and SmIIIA (IC_50_ 14 ± 2 nM) being significantly more potent than KIIIA (IC_50_ 67 ± 15 nM). A similar pattern of inhibitory potency was observed for hNa_V_1.3 and hNa_V_1.6, while no differences in potency were seen at hNa_V_1.2. At Na_V_1.1, SxIIIC and KIIIA showed similar inhibitory potency, while SmIIIA was the least potent. KIIIA, SmIIIA and SxIIIC showed minimal to no inhibition of TTX-r hNa_V_1.5 and SmIIIA and SxIIIC showed no inhibition of TTX-r hNa_V_1.8 up to 1 µM (Table 1, Figure S6). Intriguingly, µ-conotoxin inhibitory potency at hNa_V_1.7 was most varied, with a 9-fold difference between SmIIIA (IC_50_ 41 ± 4 nM) and KIIIA (IC_50_ 379 ± 43 nM) and a 4-fold difference between SmIIIA and SxIIIC (IC_50_ 152 ± 22 nM). This makes SmIIIA, in our hands, the most potent µ-conotoxin inhibitor of hNa_V_1.7 reported to date.

### 2.3. A Limited Number of µ-Conotoxins Display Inhibitory Potency at hNa_V_1.7

**Figure 2:**
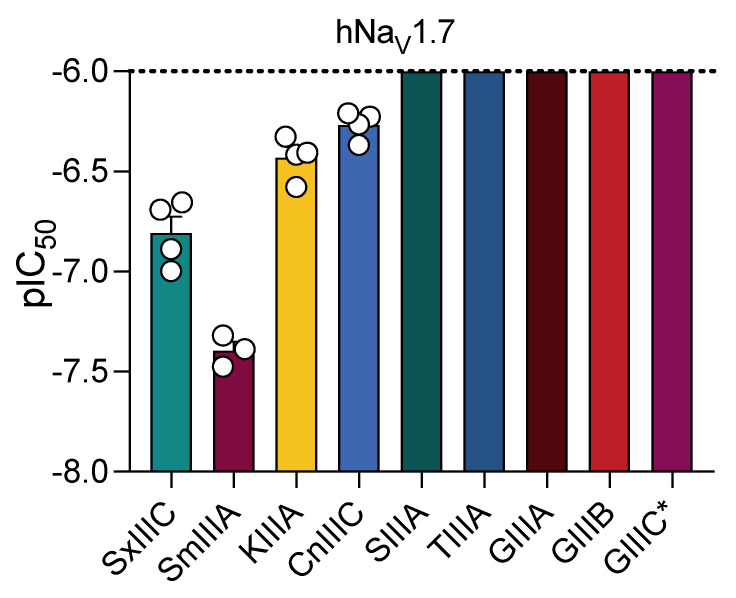
**Extended comparison of µ-conotoxins inhibiting hNa_V_1.7 assessed by whole-cell patch-clamp electrophysiology across hNa_V_1.7/β1 overexpressed in HEK293 cells**. Inhibition of hNa_V_1.7 by five additional µ-conotoxins, CnIIIC, SIIIA, TIIIA, GIIIA and GIIIB, was evaluated by automated whole-cell patch-clamp methods as described above. CnIIIC was the only additional µ-conotoxin to inhibit hNa_V_1.7 current. SIIIA, TIIIA, GIIIA and GIIIB displayed no activity up to the highest concentration tested (1 µM). GIIIC was previously found to be inactive at hNa_V_1.7 at 1 µM [26] * denotes data from references). Data are presented as mean ± SEM with individual data points (*n* = 3–4).

One of the challenges in comparing µ-conotoxin inhibitory potency between different studies is the differences in methods and species (human, mouse, and rat) used. While several µ-conotoxins have been evaluated at hNa_V_1.7 channels [4,23,24,25], only a few studies have directly compared µ-conotoxin inhibitory potency at hNa_V_1.7 in the same cell background. Therefore, we expanded our screen to analyse the hNa_V_1.7 inhibition of other conotoxins by automated whole-cell patch-clamp electrophysiology. CnIIIC inhibited hNa_V_1.7 with similar potency to KIIIA (IC_50_ 546 ± 42 nM), whereas TIIIA, GIIIA, GIIIB and SIIIA showed no inhibitory potency at hNa_V_1.7 up to concentrations of 1 µM (Figure 2). These results are consistent with previous studies where TIIIA and GIIIB (3 µM), assayed using two-electrode voltage-clamp electrophysiology, were inactive at hNa_V_1.7 [25], and SIIIA (10 µM) only produced ~75% block at hNa_V_1.7 when evaluated using whole-cell patch-clamp methods [24].

### 2.4. Charged Residues in Loop 3 Affect Selectivity for hNa_V_1.7

**Figure 3:**
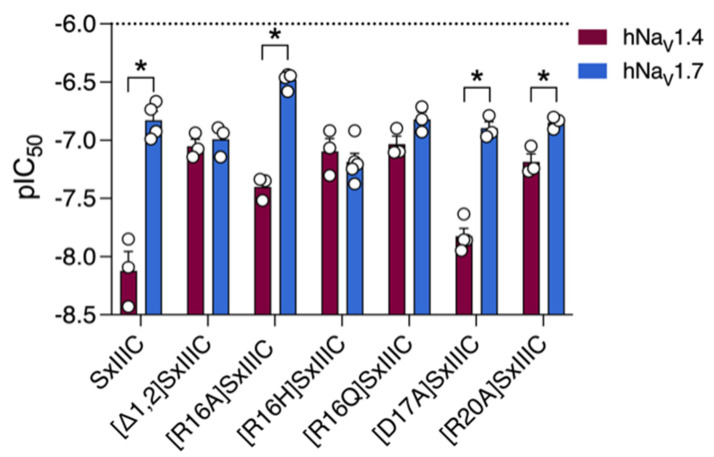
**Effect of single SxIIIC mutations on potency and selectivity at hNa_V_1.7.** pIC_50_ values ± SEM are displayed for hNa_V_1.4 (maroon bars) and hNa_V_1.7 (blue bars). [R16A]SxIIIC, [D17A]SxIIIC and [R20A]SxIIIC maintained selectivity for hNa_V_1.4 over hNa_V_1.7. In contrast, the mutations ([Δ1,2], [R16H], [R16Q]) did not affect inhibitory potency at hNa_V_1.7 but reduced potency at hNa_V_1.4, resulting in no significant selectivity between the subtypes. Data are presented as mean ± SEM with individual data points (*n* = 3–5). Statistical significance was determined by unpaired *t*-test (* *p*-value < 0.05).

Interestingly, except for SIIIA, all µ-conotoxins that potently inhibit hNa_V_1.7 contain five residues within loop 3 (the region between the fourth and fifth cysteine residues), whereas those that were inactive only contained four residues (Table 2). We were therefore interested in investigating the role of the charged residues within this loop in hNa_V_1.7 inhibition and used SxIIIC as a scaffold to design analogues with either charge-conserving or charge-neutralising substitutions (Table S1). We used SxIIIC in preference over the more active SmIIIA µ-conotoxin due to the ease of synthesis of SxIIIC compared to SmIIIA, which has proved challenging to fold. Whole-cell patch-clamp electrophysiology experiments comparing inhibitory potency of the mutants at Na_V_1.7 and Na_V_1.4 (Figure 3) showed that replacement of Arg16 by Ala, [R16A]SxIIIC, resulted in a two-fold loss of inhibition (IC_50_ 334.1 ± 25.5 nM), whereas replacement of Arg16 with a neutral sidechain in [R16Q]SxIIIC did not significantly affect the potency of SxIIIC (Table 3). Interestingly, substituting Arg 16 with His [R16H]SxIIIC (His carries a neutral charge at physiological pH due to its low pKa value, 6.0) we observed a three-fold improvement in inhibition of hNa_V_1.7 (IC_50_ 66.5 ± 2.5 nM) compared to SxIIIC. We also assessed the neighbouring residue Asp17 and observed no significant change in inhibition with the Ala-replacement analogue [D17A]SxIIIC (IC_50_ 123.5 ± 18.1 nM). Surprisingly, we also observed no difference for the Arg20 analogue [R20A]SxIIIC (IC_50_ 142.3 ± 12.9 nM), despite the analogous residue in KIIIA (Arg 14—KIIIA numbering) having previously been reported to shift subtype selectivity in favour of hNa_V_1.7 [8].

We next evaluated the inhibitory potency of these analogues, along with truncated SxIIIC, at hNa_V_1.4 to observe differences in subtype selectivity. Compared to SxIIIC (IC_50_ 12.8 ± 13.0 nM), the R16H and R16Q mutations significantly reduced potency at hNa_V_1.4 (IC_50_ 80.3 ± 24.7 nM and 99.5 ± 12.9 nM, respectively). Interestingly, this reduction gave IC_50_ values equipotent to hNa_V_1.7 (Figure 3). The same trend was observed for truncated [~1,2]SxIIIC and the [R20A]SxIIIC analogue, with both displaying reduced potency at hNa_V_1.4 (IC_50_ 96.3 ± 13.0 nM and 64.8 ± 15.4 nM, respectively) but retaining inhibitory potency at hNa_V_1.7. Although [R16A]SxIIIC slightly lost potency at hNa_V_1.4 (IC_50_ 64.8 ± 15.4 nM; Table 3), this did not affect subtype selectivity (Figure 3). In contrast, the D17A mutation in SxIIIC did not affect inhibition of either hNa_V_1.7 or hNa_V_1.4 (IC_50_ 14.6 ± 7.7 nM). Together these results reveal that several SxIIIC residues, including Arg1, Gly2, Arg16 and Arg20, may contribute to selectivity between hNa_V_1.4 and hNa_V_1.7.

### 2.5. Double and Triple SxIIIC Mutants

**Figure 4:**
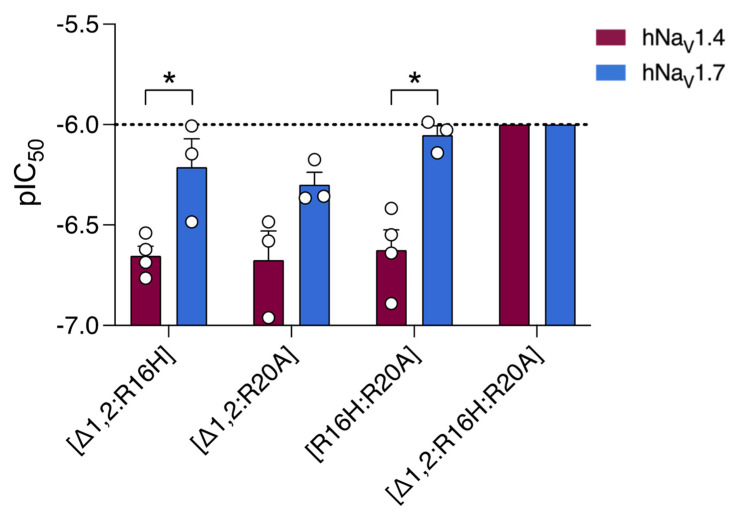
**Effect of double and triple SxIIIC mutations on potency and subtype-selectivity at hNa_V_1.4 and hNa_V_1.7.** Comparative potency of analogues displayed for hNa_V_1.4 (maroon bars) and hNa_V_1.7 (blue bars). [Δ1,2; R16H] and [R16H:R20A] maintained selectivity for hNa_V_1.4 over hNa_V_1.7. The triple mutant [Δ1,2; R16H; R20A] lost inhibitory potency. Data are presented as mean ± SEM with individual data points (*n* = 3–4). Statistical significance was determined by unpaired *t*-test (* *p*-value < 0.05).

Given the minimal disruption to hNa_V_1.7 potency caused by some SxIIIC mutations, we designed and synthesised double and triple mutant SxIIIC analogues (Table S1). Compared to native SxIIIC, all four analogues lost significant inhibitory potency at both subtypes (Figure 4). At hNa_V_1.7, the double mutants [Δ1,2; R16H], [Δ1,2; R20A] and [R16H; R20A]SxIIIC lost 4-, 3- and 6-fold potency, respectively (Table 3), whereas at hNa_V_1.4, the difference was much larger, with all three analogues decreasing potency by an average of 18-fold (Table 3). Notably, the triple mutant [Δ1,2; R16H; R20A] only displayed ~50% inhibition at hNa_V_1.7 at 1 µM and was not active at hNa_V_1.4 at 1 µM, the highest concentration tested (Figure 4 and Figure S7).

### 2.6. Key Structural Differences between hNa_V_1.7 Inhibiting µ-Conotoxins

**Figure 5:**
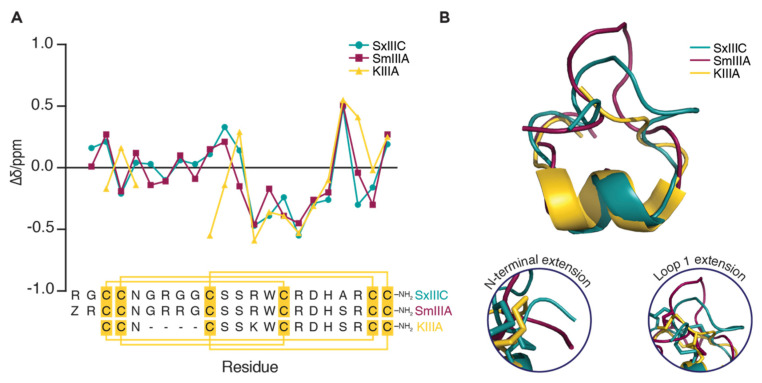
**Structural comparison of µ-conotoxins SxIIIC, SmIIIA and KIIIA.** (**A**) Comparison of secondary Hα-chemical shifts compared to random coil values [28] for three µ-conotoxins SxIIIC, SmIIIA and KIIIA [29]. Sequence alignment shows high sequence homology between µ-conotoxins with disulfide connectivity (yellow lines). (**B**) Representative 3D NMR structures (PDB: SxIIIC 6X8R, SmIIIA 1Q2J and KIIIA 2LXG) of µ-conotoxins superimposed across residues Cys10–Cys22 (SxIIIC numbering; RMSD = 1.146 Å) shows how the conserved C-terminal residues contribute to similar backbone fold, whereas the N-terminal and loop 1 extensions (inserts) extend the structures of SxIIIC and SmIIIA.

The main sequence differences of SxIIIC and SmIIIA compared to KIIIA are, a two-residue N-terminal extension and a four-residue loop 1 extension (Figure 5A). To explore the significance of these extensions, we compared the NMR solution structures (PDB: SxIIIC 6X8R, SmIIIA 1Q2J and KIIIA 2LXG). Notably, while the cysteine framework of ‘native’ KIIIA [27] differed from SxIIIC and SmIIIA, this does not appear to affect the overall structure of these µ-conotoxins. Comparison of secondary Hα chemical shift deviations from random coil values showed similar shifts between residues Arg13–Ala20 (Figure 5A), indicating conserved structural features across this region. The N-terminal half of the µ-conotoxins displayed the most variation. Backbone alignment of 3D NMR structures between Cys10 and Cys22 (SxIIIC numbering; Figure 5B) showed that the extra residues in loop 1 of SxIIIC and SmIIIA extend the top half of the structure. We propose that this section contributes to the increased potency of these two µ-conotoxins at hNa_V_1.7. Furthermore, a substitution at Ala19 in SxIIIC to Ser19 in SmIIIA and KIIIA did not appear to affect the shifts for this residue. However, the neighbouring residue Arg20 (SxIIIC numbering) did show differences between the three µ-conotoxins and suggesting a different side-chain orientation of this residue.

### 2.7. N-Terminal Deletion Does Not Significantly Affect the Potency of SxIIIC

**Figure 6:**
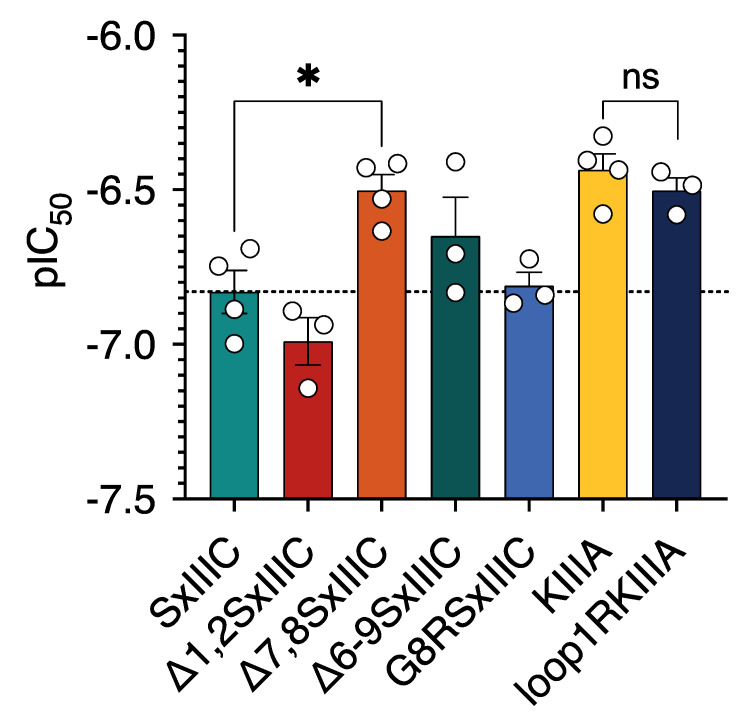
**Effects of N-terminal and loop 1 mutations on µ-conotoxin potency at hNa_V_1.7.** Comparative potency of analogues at hNa_V_1.7. N-terminal deletions of SxIIIC ([Δ1,2]SxIIIC) and the addition of loop 1 residues ([loop1R]KIIIA) into KIIIA did not significantly affect potency compared to native µ-conotoxins. Loop 1 deletions of SxIIIC ([Δ7,8] significantly reduced potency of SxIIIC, similar to that of KIIIA. Introduction of an additional charged residue into SxIIIC loop 1 ([G8R]SxIIIC) did not alter potency compared to native SxIIIC. Data are presented as mean ± SEM with individual data points (*n* = 3–4). Statistical significance for SxIIIC analogues was determined by ordinary one-way ANOVA with Dunnett’s multiple comparisons test compared to native SxIIIC (* *p*-value < 0.05). Statistical significance for KIIIA and loop1 RKIIIA was determined by unpaired *t*-test (* *p*-value < 0.05).

To assess the effects of N-terminal extensions observed in SxIIIC and SmIIIA compared to KIIIA, we designed the truncated SxIIIC analogue [Δ1,2]SxIIIC (Table S1). Due to difficulties synthesising native and modified SmIIIA, only SxIIIC and KIIIA analogues were pursued. In whole-cell patch-clamp electrophysiology experiments, we found that deletion of the N-terminal residues of SxIIIC did not significantly affect potency at hNa_V_1.7 (IC_50_ 106.2 ± 19.4 nM) compared to native SxIIIC (IC_50_ 152.2 ± 26.8 nM) (*p* > 0.05) (Figure 6).

### 2.8. Loop 1 Truncation Reduces Potency of SxIIIC at hNa_V_1.7

Interestingly, when we deleted residues within loop 1 of SxIIIC ([Δ7,8]SxIIIC), we observed reduced potency (IC_50_ 313.6 ± 36.6 nM; Table 3) compared to native SxIIIC. As these IC_50_ values were similar to KIIIA (IC_50_ 383.5 ± 15.2 nM; Figure 6), which has a similarly truncated loop 1, these results suggest that the additional loop 1 residues in SxIIIC are required for increased inhibition of hNa_V_1.7 compared to KIIIA. Furthermore, when we introduced an extra charged residue into loop 1 of SxIIIC ([G8R]SxIIIC) to mimic SmIIIA, we did not observe a significant change in potency (IC_50_ 155.7 ± 18.9 nM; Table 3) compared to SxIIIC. This observation suggests that the additional charged residue in loop 1 of SmIIIA does not cause the 4.4-fold difference in inhibition of hNa_V_1.7 observed between SxIIIC and SmIIIA. Unfortunately, previously mentioned difficulties in producing SmIIIA limited our ability to confirm this using SmIIIA analogues. Finally, when modifying KIIIA to include additional residues within loop 1 ([loop1R]KIIIA), we expected this analogue to mimic the inhibitory potency of SxIIIC. However, we observed no significant change in potency (IC_50_ 305.1 ± 23.9 nM; Table 3) compared to native KIIIA, suggesting that either the disulfide connectivity of KIIIA is unfavourable for loop 1 extensions, or that this peptide binds in a different orientation.

## 3. Discussion

µ-Conotoxins are potent inhibitors of disease-relevant Na_V_ channels, yet their promiscuous activity across different subtypes has hindered their potential for therapeutic development, as off-target activity may lead to undesirable side effects. Due to an apparent lack of inhibitory potency of many µ-conotoxins at hNa_V_1.7, only a few studies have analysed structure–activity relationships at this subtype. Here, we identified key features of µ-conotoxins of SxIIIC, SmIIIA and KIIIA that contribute to inhibition of hNa_V_1.7, including loop length, and single point mutations that affect selectivity towards hNa_V_1.4 without affecting potency at hNa_V_1.7.

We initially assessed two µ-conotoxins, SmIIIA and KIIIA, by whole-cell patch-clamp methods, to evaluate their selectivity profiles for hNa_V_ channel subtypes. Compared to SxIIIC [9], these highly homologous µ-conotoxins displayed distinct selectivity profiles (Table 4). Interestingly, our results are not in agreement with mammalian selectivity profiles reported previously for SmIIIA [20] or KIIIA [12,30]. While these inconsistencies may be attributed to the use of different techniques (two-electrode voltage- or patch-clamp methods on oocytes vs. mammalian cells; manual vs. automated techniques), or the presence of different auxiliary β-subunits, the most plausible explanation is species differences (human, rat or mouse). The earlier studies used rat isoforms, as opposed to the human subtypes used in this study. Despite the high sequence homology between species, it is not uncommon for µ-conotoxins to display different inhibitory potency across species. Such interspecies variability highlights the need to study human isoforms when evaluating the therapeutic potential of µ-conotoxins. We showed that SmIIIA inhibits hNa_V_1.7 more potently than both SxIIIC (~4-fold) and KIIIA (~10-fold). In our hands, SmIIIA is the most potent µ-conotoxin inhibitor of hNa_V_1.7 (IC_50_ 41 ± 4 nM) reported to date.

**Figure 7:**
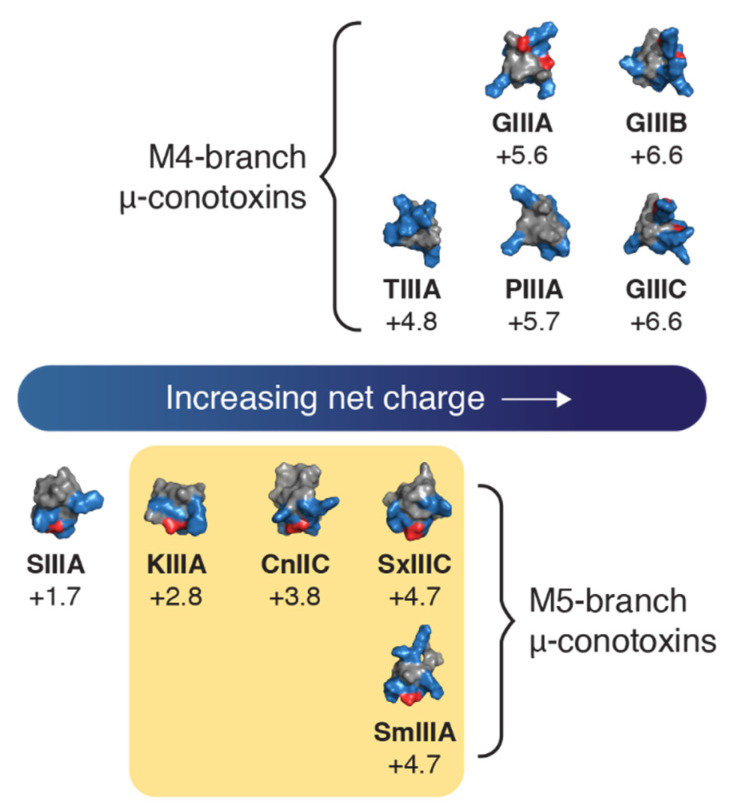
**Representation of the surface profile of µ-conotoxins and corresponding net charge.** Colours represent charged residues (basic–blue; acidic–red; neutral–grey). µ-Conotoxins active against hNa_V_1.7 (yellow shaded box) appear on the M5-branch and have an overall net charge of between +2.8–4.7.

As only a limited number of µ-conotoxins have been assessed at human Na_V_ isoforms, we explored the ability of µ-conotoxins GIIIA, GIIIB, TIIIA, CnIIIC and SIIIA to inhibit hNa_V_1.7 by whole-cell patch-clamp methods. CnIIIC was found to be equipotent with KIIIA, whereas a lack of inhibitory potency was observed for the other four µ-conotoxins at the highest concentration tested (1 µM). The results for GIIIA, GIIIB and TIIIA were consistent with the homologous µ-conotoxin GIIIC, previously shown to be inactive at hNa_V_1.7 [23]. While the results of SIIIA were expected [24], it is interesting that SIIIA (at 10 nmol, corresponding to 0.7 mg/kg) can produce an analgesic effect in a murine model of inflammatory pain [31]. Leipold et al. [3] introduced the Domain II pore loop of hNa_V_1.7 into rNa_V_1.4 and identified Asn889 (hNa_V_1.7) as responsible for the reduced sensitivity of hNa_V_1.7 to SIIIA. The corresponding residue in mNa_V_1.7 is conserved, suggesting that this residue alone may not be responsible for species differences. Another possible explanation for the lack of activity of SIIIA at hNa_V_1.7 may be the apparent low net charge (+1.7) compared to hNa_V_1.7 inhibiting µ-conotoxins, which all have a net charge of +2.8 to +4.7 (Figure 7). While µ-conotoxins share key similarities such as a conserved disulfide network, the backbone sequences between these cysteines, referred to as loops 1–3, are varied. For example, GIIIA, GIIIB, GIIIC, PIIIA and TIIIA all have four residues in loop 3 (between Cys4–Cys6) and are classed in the M4-branch µ-conotoxins [32]. In contrast, SIIIA, KIIIA, CnIIIC, SmIIIA and SxIIIC have five residues in loop 3 and are classified as M5-branch µ-conotoxins [32]. M4-branch µ-conotoxins have an increased overall net charge (≥+4.8) compared to M5-branch µ-conotoxins (Figure 7), suggesting that overall net change may contribute to µ-conotoxins’ ability to inhibit hNa_V_1.7.

To explore the role of residues in loop 3, we designed a series of µ-conotoxin analogues, opting to base our designs on SxIIIC, as opposed to the more potent SmIIIA, due to difficulties in folding the latter µ-conotoxin. Our experience in synthesising SmIIIA is consistent with literature that reports synthetic production of SmIIIA often yields multiple isomers when folded under thermodynamic conditions [33]. In the current study we used a regioselective approach for SmIIIA synthesis to ensure desired disulfide connectivity; however, despite successful direction of the disulfide bonds during regioselective SmIIIA synthesis, the yield was low, and purification proved surprisingly challenging considering the high sequence homology with SxIIIC. In contrast, SxIIIC could easily be oxidised thermodynamically, resulting in a single major isomer, or using regioselective oxidation. In addition to targeted Ala-mutations in SxIIIC, in lieu of an Ala-walk (replacing all non-Cys and Gly residues with Ala), which has been conducted for other µ-conotoxins [12,25,34,35], we were interested in exploring the consequences of different chemical properties at specific residues and thus employed rational design for our SxIIIC analogues.

Replacement of Arg16 of SxIIIC with an Ala ([R16A]SxIIIC) reduced potency two-fold at hNa_V_1.7. Our results are in contrast to those of McArthur and colleagues, who found Ala-replacement of the equivalent residue in KIIIA ([R10A]KIIIA) increased inhibition at hNa_V_1.7 [8]. That study did not report the disulfide connectivity of KIIIA, and activity studies lacked β-subunit expression, which has since been shown to affect the affinity of µ-conotoxins to Na_V_ channels [21]. Interestingly, we observed improved potency at hNa_V_1.7 and decreased inhibition at hNa_V_1.4 with a SxIIIC His-substitution at position 16, [R16H]SxIIIC. Whilst His theoretically carries a neutral charge at physiological pH, the charge state of this amino acid can be highly dependent on the environment created by surrounding amino acids and may carry a positive charge, which together with the additional bulk of the amino acid compared to the native Arg could explain the improved potency at Na_V_1.7. Another analogue, [R20A]SxIIIC, also did not affect hNa_V_1.7 inhibition and lost potency at hNa_V_1.4; however, the peptide did not display altered subtype selectivity. These results are also in contrast to Arg20 KIIIA mutations previously reported [8] that shifted selectivity towards hNa_V_1.7 from rNa_V_1.2 and rNa_V_1.4.

Using the three SxIIIC analogues that did not affect inhibition at hNa_V_1.7 but reduced potency at hNa_V_1.4, we designed and synthesised a series of double and triple mutants. None of the multiple SxIIIC mutant analogues improved potency at either channel tested (hNa_V_1.4 and hNa_V_1.7) or shifted selectivity in favour of hNa_V_1.7. While these results were disappointing, they were not completely unexpected. µ-Conotoxins have naturally evolved to potently and selectively target Na_V_ channels, and the manipulation of such an evolutionarily refined natural product can be challenging. However, one explanation for the loss in potency may have been in choosing the incorrect isomer following thermodynamic oxidation (Figures S4 and S5). To assess if the folding contributed to the loss in inhibitory potency, we additionally tested the first eluting peak for each of the analogues against hNa_V_1.4 and found them to be less active than the second eluting peak (Figure S7). In summary, none of the multiple mutant analogues improved the potency or selectivity of SxIIIC.

Given the potential clinical applications of selective Na_V_1.7 inhibition, we compared the NMR structures of three µ-conotoxins with potent inhibition of hNa_V_1.7, i.e., SxIIIC, SmIIIA and KIIIA. We identified two major differences, in the form of extensions in the N-terminal and loop 1 residues of SxIIIC and SmIIIA, both absent in KIIIA. As many residues within the highly conserved C-terminal region of the µ-conotoxins have been shown to be critical for µ-conotoxin inhibition [8,12,13,36], we proposed that these structural differences observed in the N-terminal region influenced the differences in the inhibitory potency at hNa_V_1.7. To evaluate this, we designed an additional series of µ-conotoxins analogues using SxIIIC.

By creating a truncated N-terminal analogue, we found that the N-terminal residues of SxIIIC are not essential for inhibition of hNa_V_1.7. However, truncated SxIIIC, which maintained inhibitory potency at hNa_V_1.7, compared to native SxIIIC, displayed decreased potency at hNa_V_1.4. The N-terminal extension of SxIIIC, and potentially SmIIIA, is likely to be essential for inhibition at other subtypes, as seen with µ-conotoxin KIIIB (identical to KIIIA but with a two-residue N-terminal extension) which inhibits rNa_V_1.2 with 5-fold greater affinity than KIIIA [27]. These results are consistent with truncated SIIIA, which showed that these N-terminal residues played a non-essential role in rNa_V_1.2 inhibition [34]. It could be suggested that these N-terminal residues play a role in guiding the µ-conotoxin to the binding site in different Na_V_ channel subtypes. In contrast to the N-terminal extension, we discovered that residues within loop 1 of SxIIIC influenced inhibition of hNa_V_1.7. When loop 1 residues were removed to mimic KIIIA, the potency of SxIIIC reverted to IC_50_ values similar to KIIIA. As many studies interested in optimising KIIIA for Na_V_1.7 inhibition have focused on downsizing or reducing the µ-conotoxins to only retain residues contributing to the pharmacophore [14,15,16], our findings identify for the first time the importance of the residues within loop 1 of SxIIIC for potent inhibition of hNa_V_1.7. Interestingly, when we reintroduced extra loop 1 residues into KIIIA, we did not observe the expected improved potency. By revisiting the NMR structure, we observed that although KIIIA had a different disulfide connectivity to SxIIIC, the overall conformation of the disulfide bonds did not change. This suggests that KIIIA with I–V, II–IV, III–VI connectivity might not be optimal for the addition of residues in loop 1 and this could be explored in future studies.

In addition to improved selectivity for Na_V_1.7, which is essential to avoid severe systemic side effects, species selectivity of the µ-conotoxins currently limits preclinical efficacy test in relevant rodent models of pain. Accordingly, future work, such as modelling or structural studies using relevant channel states and subtypes, will be required to facilitate drug development efforts, although oral delivery of peptides remains a challenge that would likely limit µ-conotoxin-based therapeutics to parenteral administration.

In conclusion, we have identified that the number of residues within loops 1 and 3 of SxIIIC, SmIIIA and KIIIA contributes to potency at hNa_V_1.7. Overall, the findings of this study provide valuable information for future design of hNa_V_1.7 specific µ-conotoxin derived inhibitors.

## 4. Methods

### 4.1. Peptide Synthesis

KIIIA, SIIIA, SxIIIC and analogues including [Δ1,2]SxIIIC, [R16A]SxIIIC, [R16H]SxIIIC, [R16Q]SxIIIC, [D17A]SxIIIC, [R20A]SxIIIC, [R20E]SxIIIC, [R20H]SxIIIC, [R20Q]SxIIIC, [R20W]SxIIIC, [Δ1,2:R16H]SxIIIC [Δ1,2:R20A]SxIIIC, [R16H:R20A]SxIIIC and [Δ1,2:R16H:R20A]SxIIIC) were synthesised using methods described earlier [9]. Protecting groups trityl (Trt), acetamidomethyl (Acm) and 4,4′-dimethylsulfinylbenzhydryl (Msbh) were used for regioselective protection of SmIIIA and SxIIIC analogues ([Δ7,8]SxIIIC, [Δ6–9]SxIIIC and [G8R]SxIIIC) with connectivity Cys3–Cys15(Msbh)/Cys4–Cys21(Acm)/Cys10–Cys22(Trt). The KIIIA analogue [loop1R]KIIIA was produced with connectivity Cys1–Cys15(Msbh)/Cys2–Cys9(Acm)/Cys4–Cys16(Trt). All peptides were assembled on a CEM Liberty Prime automatic synthesiser (CEM, Matthews, NC, USA) using rink amide-AM resin. Peptides were cleaved from solid support and side chains simultaneously deprotected in 92.5% trifluoroacetic acid (TFA)/2.5% triisopropylsilane/2.5% water/2.5% 2,2-(ethylenedioxy)diethanethiol for 2 h at room temperature. Excess TFA acid was evaporated by N_2_ flow, followed by peptide precipitation in ice-cold diethyl ether and centrifugation. Peptides were redissolved in solvent B (50% acetonitrile (ACN)/0.01% TFA) and lyophilised. TIIIA, GIIIA and GIIIB were synthesised as previously described [25,34]. A list of peptide sequences can be found in Supplementary Materials (Table S1).

### 4.2. Oxidation

Most SxIIIC analogues were oxidised thermodynamically to form disulfide bonds as described earlier [9]. However, due to difficulties obtaining the desired connectivity using thermodynamic oxidation, some peptides were oxidised using orthogonal protection of pairs of Cys residues. This regioselective oxidation was done as described previously [26,37], whereby linear peptides with Trt group removed were dissolved in acetic acid (2 mg/mL) and stirred at room temperature. To oxidise the first Cys pair, one equivalent of I_2_ (dissolved in MeOH) was added dropwise until the reaction turned pale-yellow and the colour persisted. Five equivalents of free Trp were added to minimise side-reactions with iodine. Following 15 min of stirring, the reaction was diluted with water and hydrochloric acid to a final volume of 50% acetic acid and 1% hydrochloric acid. To remove the Acm group and form the second disulfide bond, eight equivalents of I_2_ were added to the reaction and stirred for a further 30 min. The reaction was quenched with aqueous ascorbic acid until the solution became colourless. The intermediate product was isolated by RP-HPLC on a Shimadzu LC-20AT system equipped with an SPD-20A Prominence UV/VIS detector, and a FRC-10A fraction collector. Peptides were diluted to <2% acetic acid and loaded onto a Gemini, 5 µm C18 110 Å, 250 × 10 mm column (Phenomenex, Torrance, CA, USA). Peptides were purified using linear gradient between 5–25% solvent B over 40 min at 3 mL/min (solvent A: 0.05% TFA in H_2_O; solvent B: 90% ACN/0.05% TFA in H_2_O). Fractions containing the desired product were identified by electrospray ionisation-mass spectrometry (ESI-MS) and lyophilised. To remove the Msbh protecting groups and form the final disulfide bond, peptides were dissolved in TFA (1 mg/mL) and cooled on ice. Five equivalents of free Trp, dimethyl sulfate (1% *v*/*v*) and 20 equivalents of NaI were added and stirred for 15 min. The reaction was quenched by diluting the reaction with aqueous ascorbic acid (10 nM; 15 times the initial volume of TFA). The final product was isolated by RP-HPLC using a Gemini, 5 µm C18 110 Å, 250 × 5 mm column and a linear gradient 0–20% solvent B at 1 mL/min over 40 min. Fractions containing the desired product were identified by ESI-MS (Shimadzu LCMS-2020), lyophilised and stored at −20 °C. For analytical RP-HPLC and ESI-MS of the final products, see Figures S1–S4.

### 4.3. Cell Culture

HEK293 cells heterologously expressing hNa_V_1.1–1.7/β1 (SB Drug Discovery, Glasgow, UK) or CHO cells heterologously expressing hNa_V_1.8/β3 in a tetracycline-inducible system (ChanTest, Cleveland, OH, USA) were maintained in Minimum Essential Medium Eagle (M5650) supplemented with 10% *v*/*v* foetal bovine serum, 2 mM L-glutamine, and the selection antibiotics as recommended by the manufacturer. Cells were incubated at 37 °C with 5% CO_2_ and split every 3–4 days when reaching 70–80% confluency using the dissociation reagent TrypLE Express (Thermo Fisher Scientific, Scoresby, VIC, Australia). Expression of hNa_V_1.8 was induced by the addition of tetracycline (1 μg/mL) for 48 h prior to assays.

### 4.4. Whole-Cell Patch-Clamp Electrophysiology Assays

Automated whole-cell patch-clamp recordings were performed with a QPatch-16 automated electrophysiology platform (Sophion Bioscience, Ballerup, Denmark) as previously described [9].

The extracellular solution consisted of (in mM) 145 NaCl, 4 KCl, 2 CaCl_2_, 1 MgCl_2_, 10 HEPES, and 10 glucose, pH to 7.4 with NaOH (adjusted to 305 mOsm/L with sucrose). The intracellular solution consisted of (in mM) 140 CsF, 1 EGTA, 5 CsOH, 10 HEPES, and 10 NaCl, pH to 7.3 with CsOH (adjusted to 320 mOsm/L with sucrose). Peptides were diluted in extracellular solution with 0.1% bovine serum albumin and each concentration was incubated for 5 min. hNa_V_1.1–1.7 currents were elicited by a 50 ms test pulse to −20 mV from a holding potential of −90 mV (repetition interval 20 s). hNa_V_1.8 currents were elicited by a 50 ms test pulse to +10 mV from a holding potential of −90 mV (repetition interval 20 s) in the presence of TTX (1 µM) to inhibit endogenous TTX-sensitive current in CHO cells. Recordings were taken at ambient room temperature (22 °C). Peak current post-peptide addition (I) was normalised to peak current of buffer control (I_0_). IC_50_s were determined by plotting difference in peak current (I/I_0_) and log peptide concentration. Concentration-response curves were fitted using the log (inhibitor) vs. response-variable slope (four parameters) Equation (1) to obtain pIC_50_ values for each biological replicate.

y = 1/(1 + 10^((LogIC_50_ − x) × HillSlope)) (1)

The corresponding IC_50_s were used to compute mean ± standard error of the mean from at least *n* = 3 biological replicates.

## 5. Data Analysis

Data were analysed using GraphPad Prism 9.3.1. Statistical significance, defined as *p* < 0.05, was determined using One-way ANOVA with Tukey’s multiple comparison of IC_50_ or pIC_50_ values fitted for individual biological replicates as described above.

## Figures and Tables

**Table 1:** Summary of µ-conotoxin IC_50_ values. IC_50_ values displayed in nM ± SEM.

Subtype	SxIIIC ^a^	SmIIIA	KIIIA
Na_V_1.1	132 ± 12	235 ± 15.0	136 ± 18
Na_V_1.2	364 ± 54	172 ± 49	186 ± 32
Na_V_1.3	89 ± 11	95 ± 9	>1000
Na_V_1.4	15 ± 11	14 ± 2	67 ± 15
Na_V_1.5	>1000	>1000	>1000
Na_V_1.6	125 ± 11	106 ± 37	762 ± 77
Na_V_1.7	152 ± 22	41 ± 4	379 ± 43
Na_V_1.8	>1000	>1000	>1000

*n* = 3–10 cells per data point. ^a^ Results from [9].

**Table 2:** Comparison of µ-conotoxin sequences.

µ-Conotoxin	Sequence	Inhibitory Potency at hNa_V_1.7
GIIIA	RD**CC**TOOKK**C**KDRQ**C**KOQ-R**CC**A *	No
GIIIB	RD**CC**TOORK**C**KDRR**C**KOM-K**CC**A *	No
GIIIC	RD**CC**TOOKK**C**KDRR**C**KOL-K**CC**A *	No
TIIIA	RHG**CC**KGOKG**C**SSRE**C**ROQ-H**CC** *	No
CnIIIC	G**CC**NGPKG**C**SSKW**C**RDHAR**CC** *	Yes
KIIIA	**CC**N—**C**SSKW**C**RDHSR**CC** *	Yes
SIIIA	ZN**CC**NG—G**C**SSKW**C**RDHAR**CC** *	No
SmIIIA	ZR**CC**NGRRG**C**SSRW**C**RDHSR**CC** *	Yes
SxIIIC	RG**CC**NGRGG**C**SSRW**C**RDHAR**CC** *	Yes

* C-terminal amidation, Z-pyroglutamic acid, O-hydroxyproline.

**Table 3:** Summary of IC_50_ values for SxIIIC analogues at hNa_V_1.7 and hNa_V_1.4. IC_50_ displayed in nM ± SEM.

Analogue	hNa_V_1.7	Fold Difference to SxIIIC	hNav1.4	Fold Difference to SxIIIC
SxIIIC	152.2 ± 26.8	−	12.8 ± 13.0	−
[Δ1,2]SxIIIC	106.2 ± 19.4	ns	96.3 ± 13.0	↓7-fold
[Δ7,8]SxIIIC	313.6 ± 36.6	↓2-fold	−	−
[Δ6–9]SxIIIC	317.9 ± 100.0	↓2-fold	−	−
[G8R]SxIIIC	155.7 ± 18.9	ns	−	−
[Loop1R]KIIIA	305.1 ± 23.9	↓2-fold *	−	−
[R16A]SxIIIC	334.1 ± 25.5	↓2-fold	38.0 ± 13.0	↓3-fold
[R16H]SxIIIC	66.5 ± 2.5	↑2-fold	80.3 ± 24.7	↓6-fold
[R16Q]SxIIIC	151.3 ± 24.0	ns	99.5 ± 12.9	↓7-fold
[D17A]SxIIIC	123.5 ± 18.1	ns	14.6 ± 7.7	ns
[R20A]SxIIIC	142.3 ± 12.9	ns	64.8 ± 15.4	↓5-fold
[Δ1,2; R16H]	666.6 ± 211.6	↓4-fold	221.4 ± 26.9	↓17-fold
[Δ1,2; R20A]	481.8 ± 646.9	↓3-fold	222.3 ± 70.4	↓17-fold
[R16H; R20A]	888.5 ± 916.4	↓6-fold	247.8 ± 57.7	↓19-fold
[Δ1,2; R16H; R20A]	>1000	−	>1000	−

*n* = 3–4 cells per data point. ns—no significant difference. * compared to KIIIA. ↓ fold-decrease, and ↑ fold-increase in potency compared to SxIIIC.

**Table 4:** Comparison of µ-conotoxin selectivity profiles of hNa_V_ channels.

µ-Conotoxin	Selectivity Profile
SxIIIC	1.4 > 1.3 > 1.6 ≈ 1.1 ≈ **1.7** > 1.2 >> 1.5 ≈ 1.8
SmIIIA	1.4 > **1.7** > 1.3 ≈ 1.6 > 1.2 > 1.1 >> 1.5 ≈ 1.8
KIIIA	1.4 > 1.1 > 1.2 > **1.7** > 1.6 > 1.3 >> 1.5 ≈ 1.8

## Data Availability

Data is contained within the article or Supplementary Materials.

## Supplementary Materials

The following supporting information can be downloaded at: https://www.mdpi.com/article/10.3390/toxins14090600/s1. Figure S1: Analytical RP-HPLC traces with corresponding ESI-MS spectra of native μ-conotoxins used in this study; Figure S2: Analytical RP-HPLC traces with corresponding ESI-MS spectra of single mutants and N-terminus truncated SxIIIC analogues used in this study; Figure S3: Crude and analytical RP-HPLC traces with corresponding ESI-MS spectra following regioselective oxidation of SxIIIC analogues used in this study; Figure S4: RP-HPLC traces repre-senting the crude oxidation of double and triple mutant SxIIIC analogues highlighting the two main folding isomers (peak 1—blue arrow, and peak 2—red arrow), and analytical RPHPLC traces of isolated fractions (peak 2—red arrow), with corresponding ESI-MS spectra of peak 2 (red arrow); Figure S5: 1D 1H NMR spectra of the amide region of the first and second peaks from the double and triple mutant SxIIIC analogues; Figure S6: Concentration response curves of A KIIIA, SmIIIA, SxIIIC at TTX-r hNaV1.5/b1 overexpressed in HEK293 cells, and B SmIIIA and SxIIIC at TTX-r hNaV1.8/b3 overexpressed in CHO cells using automated whole cell patch clamp electrophysiol-ogy; Figure S7: Concentration response curves for the two major peaks (First peak—A, blue arrow in Figure S4; Second peak—B, red arrow in Figure S4) of the double and triple mutants against hNaV1.4 as assessed by whole-cell patch-clamp electrophysiology; Table S1: Sequence table of µ-conotoxins and analogues used in this study.
