# Supplementary material for: µ-Conotoxins Targeting the Human Voltage-Gated Sodium Channel Subtype NaV1.7
Source: Toxins (Basel). 2022 Aug 30;14(9):600. doi: 10.3390/toxins14090600 (PMC9506549; doi:10.3390/toxins14090600)
Supplement: Supplementary file 1 [file toxins-14-00600-s001.zip › toxins-1841787-supplementary.pdf]

toxins

Supplementary Materials: μ-Conotoxins Targeting the Human Voltage-Gated Sodium Channel Subtype Nav1.7

Kirsten L. McMahon, Hue N. T. Tran, Jennifer R. Deuis, David J. Craik, Irina Vetter and Christina I. Schroeder

Table S1. – Sequence table of μ-conotoxins and analogues used in this study.

Name	Sequence	Theoretical mass (Da) [M+H]+	Observed mass (Da) [M+H]+
**SxIIIC**	RG **CC** NGRG**GC**SSR**WC**RDHAR **CC** *	2436.83	2435.30
**SmIIIA**	ZR **CC** NGRRG**C**SSRW**C**RDHSR **CC** *	2606.01	2607.70
KIIIA	**CC**N----**C**SSKW**C**RDHSR**CC***	1884.19	1882.85
GIIIA	RD**CC**TOOKK**C**KDRQ**C**KOQ-R**CC**A*	2609.05	2608.87
GIIIB	RD**CC**TOORK**C**KDRR**C**KOM-K**CC**A*	2640.26	2640.17
TIIIA	RHG**CC**KGOKG**C**SSRE**C**ROQH-**CC***	2457.88	2457.73
SIIIA	ZN**CC**NG--G**C**SSKW**C**RDHAR**CC***	2207.54	2207.15
CnIIIC	G**CC**NGPKG**C**SSKW**C**RDHAR**CC***	2264.65	2265.00
[Δ1,2]SxIIIC	**CC**NGRGG**C**SSRW**C**RDHAR**CC***	2223.57	2223.33
[Δ7,8]SxIIIC	RG**CC**NG--G**C**SSRW**C**RDHAR**CC***	2223.60	2223.07
[Δ6–9]SxIIIC	RG**CC**N----**C**SSRW**C**RDHAR**CC***	2109.50	2109.40
[G8R]SxIIIC	RG**CC**NGR**R**G**C**SSRW**C**RDHAR**CC***	2535.95	2536.00
[loop1R]KIIIA	**CC**N**GRGG****C**SSKW**C**RDHSR**CC***	2211.60	2211.23
[R16A]SxIIIC	RG**CC**NGRGG**C**SSRW**CA**DHAR**CC***	2351.70	2351.00
[R16H]SxIIIC	RG**CC**NGRGG**C**SSRW**CH**DHAR**CC***	2417.72	2417.37
[R16Q]SxIIIC	RG**CC**NGRGG**C**SSRW**CQ**DHAR**CC***	2408.71	2408.43
[D17A]SxIIIC	RG**CC**NGRGG**C**SSRW**C**R**A**HAR**CC***	2392.75	2392.47
[R20A]SxIIIC	RG**CC**NGRGG**C**SSRW**C**RDHA**ACC***	2351.65	2351.27
[R20H]SxIIIC	RG**CC**NGRGG**C**SSRW**C**RDHA**HCC***	2417.72	2417.37
[R20E]SxIIIC	RG**CC**NGRGG**C**SSRW**C**RDHA**ECC***	2409.69	2409.33
[R20W]SxIIIC	RG**CC**NGRGG**C**SSRW**C**RDHA**WCC***	2466.79	2466.40
[R20Q]SxIIIC	RG**CC**NGRGG**C**SSRW**C**RDHA**QCC***	2408.71	2408.43
[Δ1,2;R16H]SxIIIC	**CC**NGRGG**C**SSRW**CH**DHAR**CC***	2204.54	2203.90
[Δ1,2;R20A]SxIIIC	**CC**NGRGG**C**SSRW**C**RDHA**ACC***	2138.45	2138.17
[R16H;R20A]SxIIIC	RG**CC**NGRGG**C**SSRW**CH**DHA**ACC***	2332.67	2332.27
[Δ1,2;R16H;R20A]SxIIIC	**CC**NGRGG**C**SSRW**CH**DHA**ACC***	2119.43	2119.13

Cys residues highlighted in bold with yellow shading, mutations highlighted in bold red, Z – pyroglutamate, O – hydroxyproline, and * denotes amidated C-terminus.

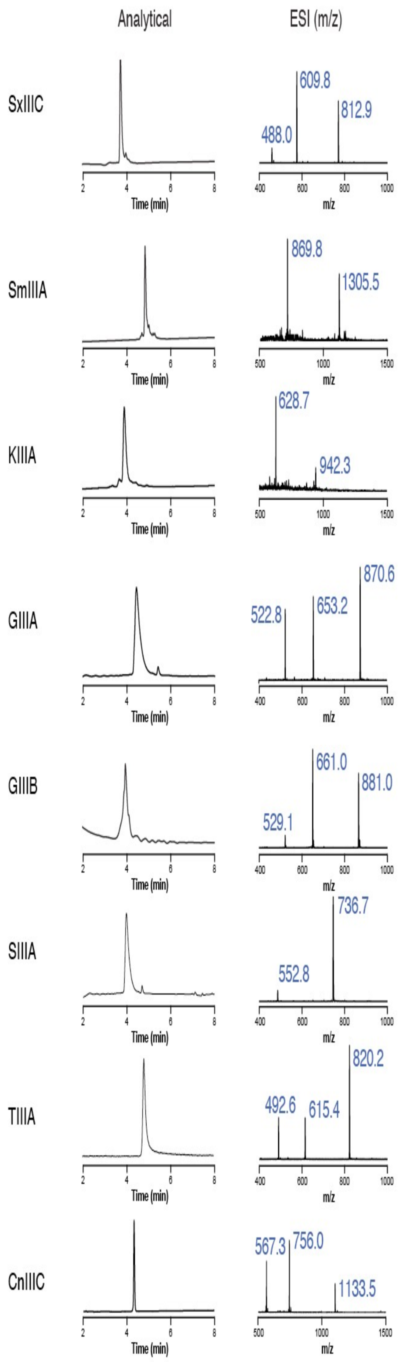

Figure S1. – Analytical RP-HPLC traces with corresponding ESI-MS spectra of native µ-conotoxins used in this study. The final product was analyzed by RP-HPLC using a Gemini, 5 µm C18 110 Å, 250 × 5 mm column and a linear gradient 0–20% solvent B at 1 mL/min over 40 min and identified by ESI-MS (Shimadzu LCMS2020), lyophilised and stored at –20 °C prior to use. Solvent A: 0.05% TFA in H_2O; solvent B: 90% ACN/0.05% TFA in H_2O.

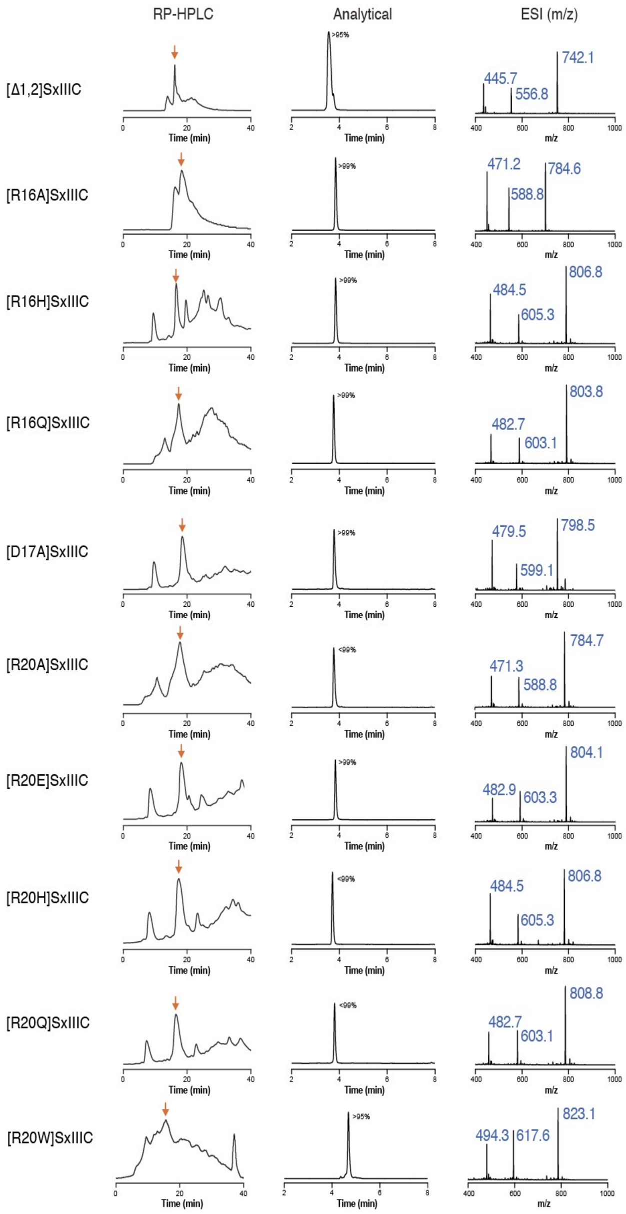

Figure S2. – Analytical RP-HPLC traces with corresponding ESI-MS spectra of single mutants and N-terminus truncated SxIIIC analogues used in this study. The final product was isolated by RP-HPLC using a Gemini, 5 µm C18 110 Å, 250 × 5 mm column and a linear gradient 0–20% solvent B at 1 mL/min over 40 min. Fractions containing the desired product were identified by ESI-MS (Shimadzu LCMS-2020), lyophilised and stored at. –20 °C). The final product was analyzed using RP-HPLC 0–100% B at 0.2 mL/min over 10 min.

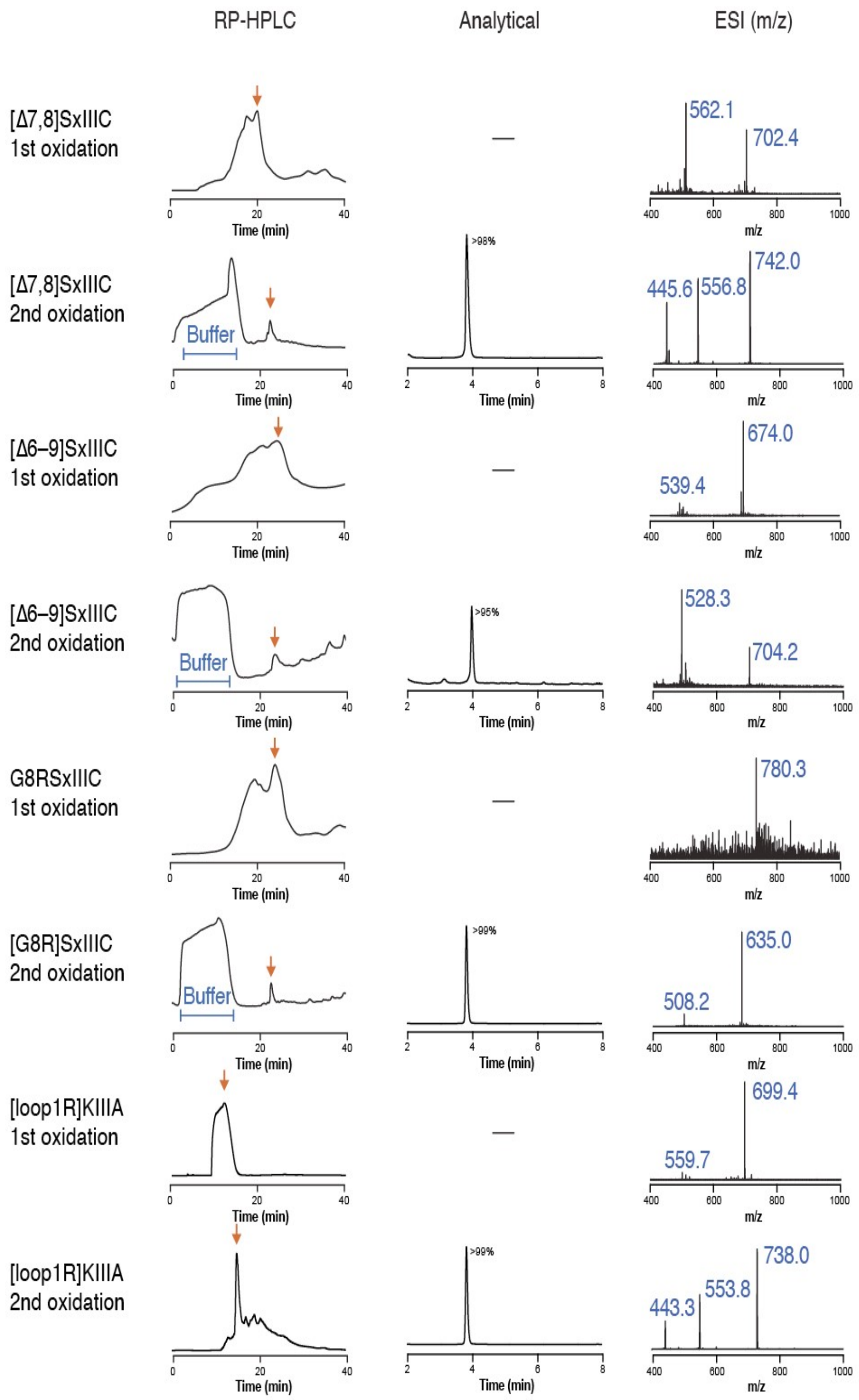

Figure S3. – Crude and analytical RP-HPLC traces with corresponding ESI-MS spectra following regioselective oxidation of SxIIIC analogues used in this study. The intermediate product was isolated by RPHPLC on a Shimadzu LC-20AT system equipped with a SPD-20A Prominence UV/VIS detector, and a FRC10A fraction collector. Peptides were purified using linear gradient between 5–25% solvent B over 40 min at 3 mL/min. Fractions containing the desired product were identified by electrospray ionisation-mass spectrometry (ESI-MS) and lyophilised. Fractions containing the desired product were identified by ESI-MS (Shimadzu LCMS-2020), lyophilised and stored at –20 °C. The final product was analyzed by RP-HPLC on a linear gradient 0–100% solvent B at 0.2 mL/min over 10 min. .

Double and triple SxIIIC analogues

In contrast to native SxIIIC following thermodynamic oxidation, the double and triple mutant analogues did not produce a single major isomer during folding rather, two equally prominent folded products (Figure S4). It is not unusual for µ-conotoxins to produce different folds (29, 35). Following one-dimensional ^{1}H spectra NMR analysis (Figure S5), the spectra for peak 2 more closely resembled native SxIIIC and was therefore selected for assays (Figure S6).

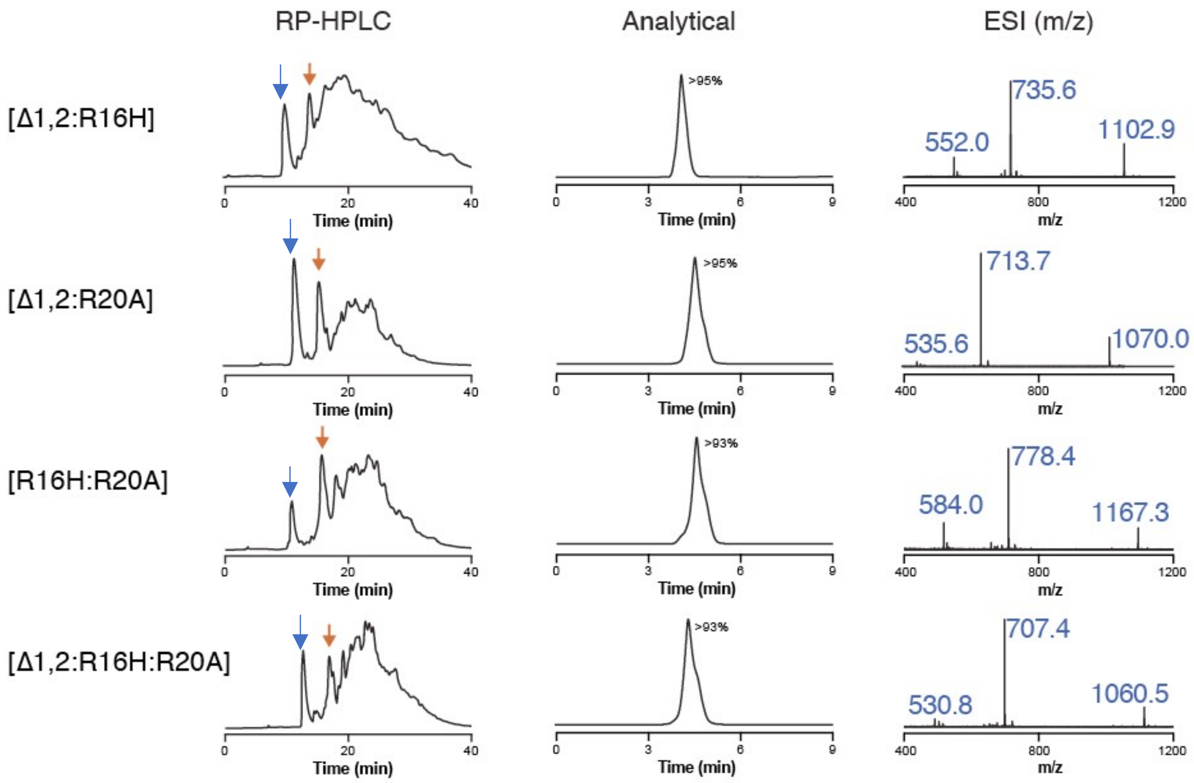

Figure S4. – RP-HPLC traces representing the crude oxidation of double and triple mutant SxIIIC analogues highlighting the two main folding isomers (peak 1 – blue arrow, and peak 2 – red arrow), and analytical RPHPLC traces of isolated fractions (peak 2 – red arrow), with corresponding ESI-MS spectra of peak 2 (red arrow). Peptides were purified using linear gradient between 5–25% solvent B over 40 min at 3 mL/min. Fractions containing the desired product were identified by electrospray ionisation-mass spectrometry (ESI-.MS) and lyophilised. Fractions containing the desired product were identified by ESI-MS (Shimadzu LCMS-2020), lyophilised and stored at –20 °C. The final product was analyzed by RP-HPLC on a linear gradient 0–100% solvent B at 0.2 mL/min over 10 min.

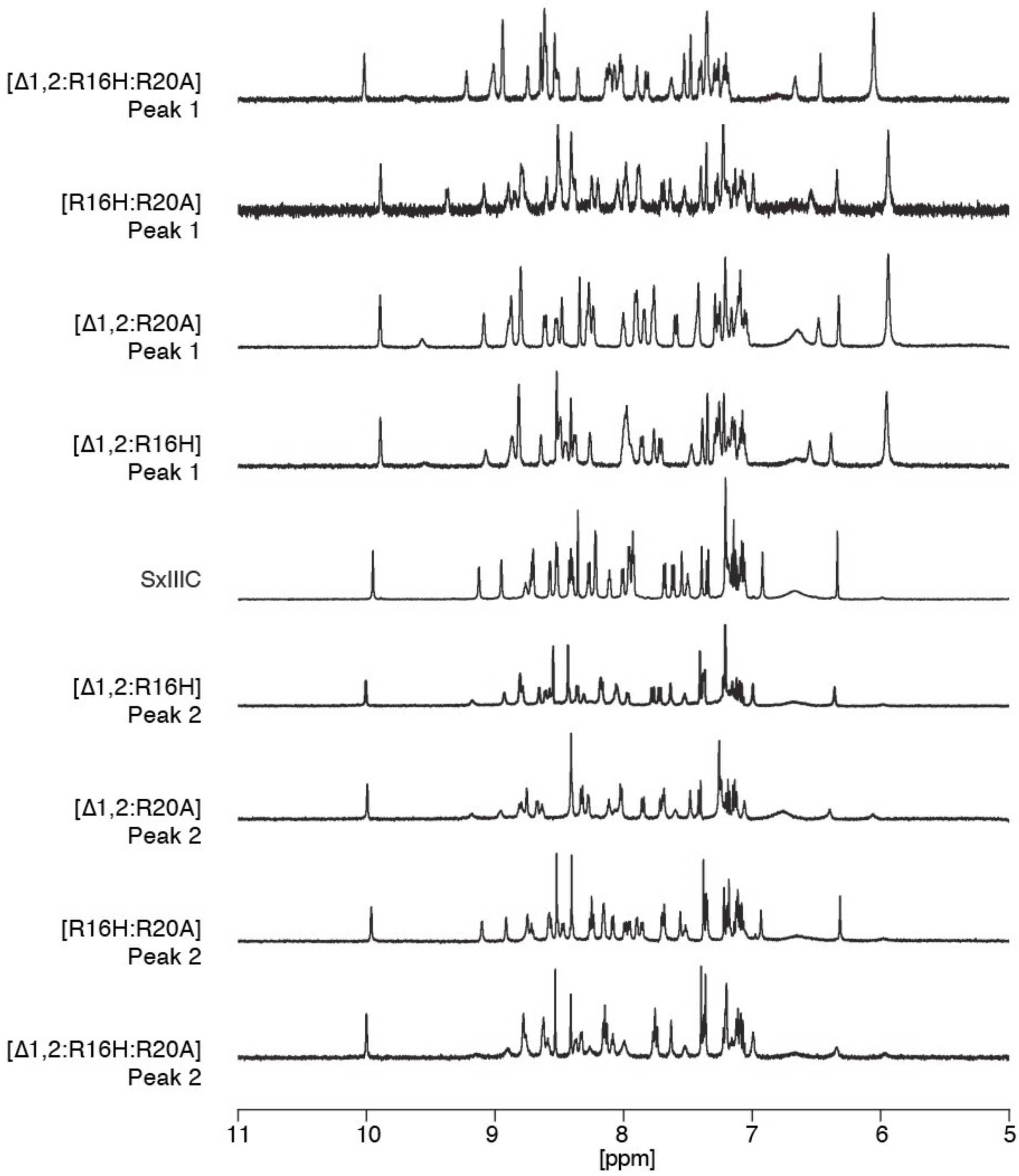

Figure S5. – 1D ^{1}H NMR spectra of the amide region of the first and second peaks from the double and triple mutant SxIIIC analogues. While peak 1 (blue arrow in Figure S4) spectra correspond to a folded peptide, the second peak (red arrow in Figure S4) more closely represents native SxIIIC and was subsequently selected for assays. NMR experiments were carried out on a Bruker Avance III 600 MHz equipped with a cryoprobe. (Bruker, Sydney, NSW, Australia). Peptides were dissolved in 90% H_2O / 10% D_2O (*v*/*v*) at 1 mg/mL. All NMR experiments were conducted at 298 K and pH 4.0.

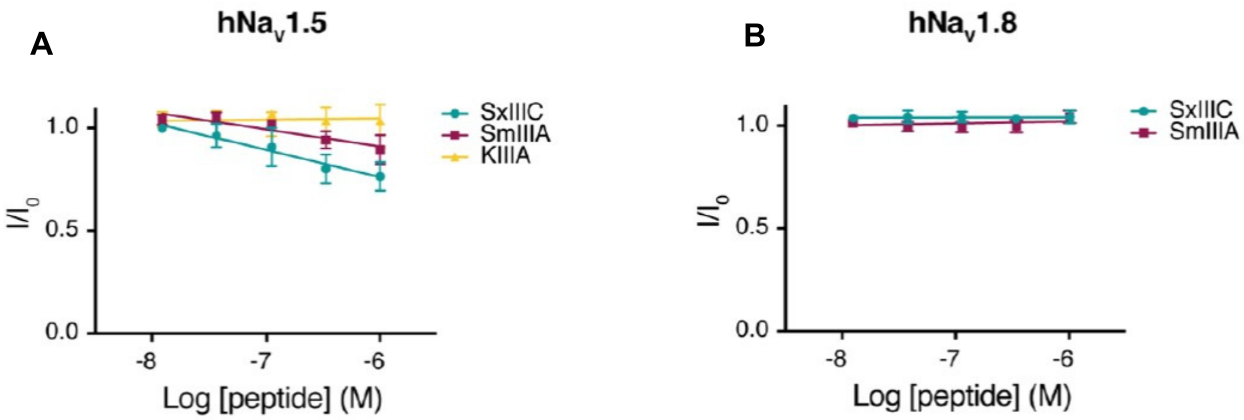

Figure S6. – Concentration response curves of **A** KIIIA, SmIIIA, SxIIIC at TTX-r hNav1.5/b1 overexpressed in HEK293 cells, and **B** SmIIIA and SxIIIC at TTX-r hNav1.8/b3 overexpressed in CHO cells using automated whole cell patch clamp electrophysiology. hNav1.5 currents were elicited by a 50 ms test pulse to −20 mV from a holding potential of −90 mV (repetition interval 20 s). hNav1.8 currents were elicited by a 50 ms test pulse to +10 mV from a holding potential of −90 mV (repetition interval 20 s) in the presence of TTX (1 μM) to inhibit endogenous TTX-sensitive current in CHO cells. Recordings were taken at ambient room temperature (22 °C). Peak current post-peptide addition (I) was normalised to peak current of buffer control (I_0). IC_{50}s were determined by plotting difference in peak current (I/I_0) and log peptide concentration.

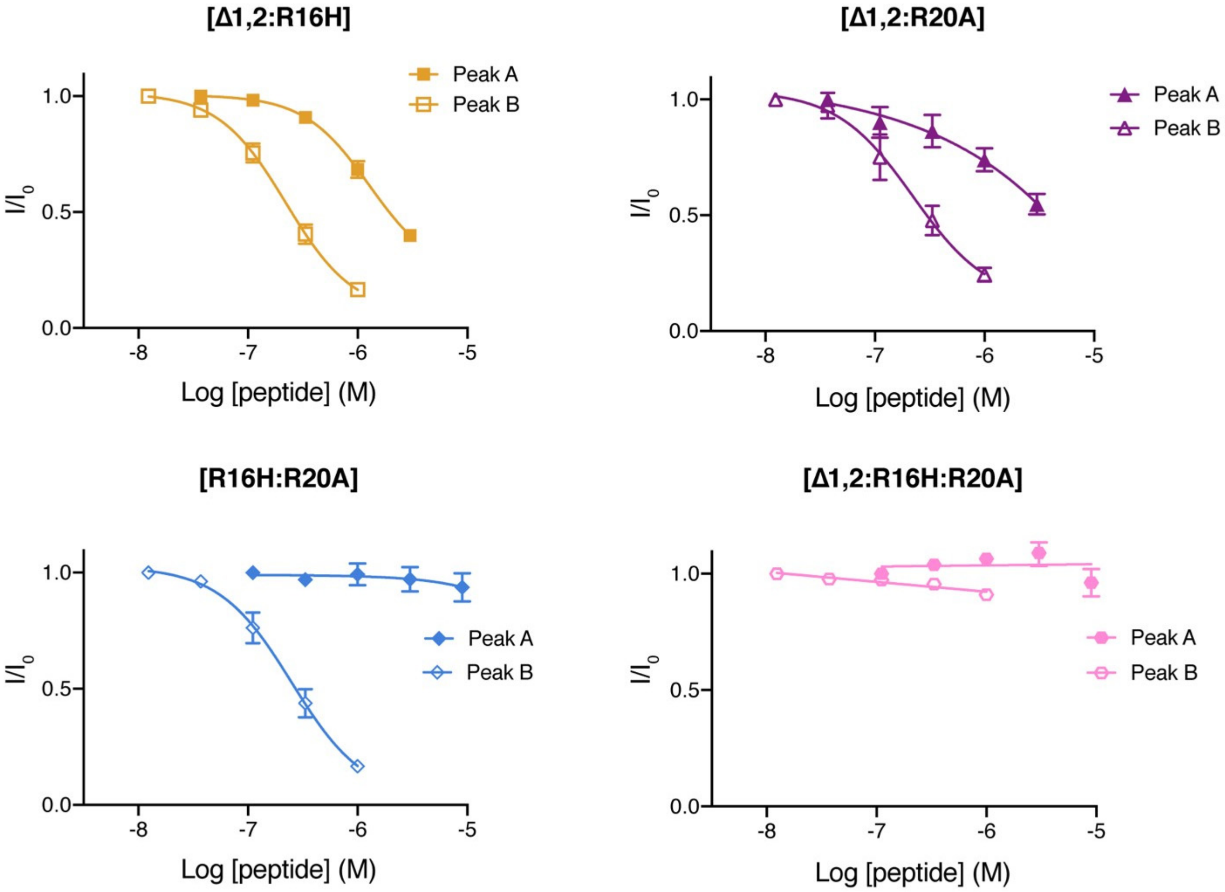

Figure S7. – Concentration response curves for the two major peaks (First peak – A, blue arrow in Figure S4; Second peak – B, red arrow in Figure S4) of the double and triple mutants against hNav1.4 as assessed by whole-cell patch-clamp electrophysiology. hNav1.4 currents were elicited by a 50 ms test pulse to −20 mV from a holding potential of −90 mV (repetition interval 20 s).

Recordings were taken at ambient room temperature (22 °C). Peak current post-peptide addition (I) was normalised to peak current of buffer control (I_0). IC_{50}s were determined by plotting difference in peak current (I/I_0) and log peptide concentration.
